# 

**Published:** 1861-04

**Authors:** 


### CHICAGO MEDICAL JOURNAL.

**Terms, $2.00 per Annum.**

### CONTENTS OF THE APRIL NUMBER.

ORIGINAL COMMUNICATIONS.

PAGE.

Application of Gutta Percha to Club Foot Dressings. By R. L. Rea, M. D., Prof. of Anat. Rush Med. College.......................... 193

Retroversion of the Uterus, with a description of an instrument designed for its reposition. By H. W. Jones, M. D....................... 199

Report of Surgical Cases. By Geo. K. Amerman, M. D., one of the attending Surgeons to the City Hospital............................ 202

The formation of Coagula in the Circulatory System. By E. L. Holmes, M. D., of Chicago. (Read before the Chicago Medical Society).... 207

Case of Irreducible Scrotal Hernia successfully treated. By Ross C. Russ, M. D., Royalton, Ind....... ................................ .... 214

Retention of Fœtus several months after its Death. By J. Tripp, M. D., Morenci, Mich............................................ 215

Proceedings of Societies.............................................. 215

SELECTED.

Treatment of Ununited Fracture by Sub-Cutaneous Perforation of Bone.. 218

Case of Hydrocephalus; Puncture; Death.......................... 221

Professor Forget on Interrogation of Patients........................ 224

On the Action of Iodine of Iron upon Phthisis. By Dr. Cotton, Physician to the Hospital for Consumption at Brompton................... 225

Slow Poisoning by Preparations of Lead, and its influence on the product of conception. By Constantine Paul,—Arch. Gen. de Med., May, 1860 227

Finger-Nail Signs.................................................. .. 228

Galactics ......................................................... 229

EDITORIAL.

Editorial and Miscellaneous............................................ 230

Mortality of Chicago for February, 1861............................ 254

Medical Works....................................... .............. 256

